# Standardised unfold map of the left atrium: regional definition for multimodal image analysis

**DOI:** 10.1186/1532-429X-17-S1-P41

**Published:** 2015-02-03

**Authors:** Catalina Tobon-Gomez, Maria A Zuluaga, Henry Chubb, Steven E Williams, Constantine Butakoff, Rashed Karim, Oscar Camara, Sebastien Ourselin, Kawal Rhode

**Affiliations:** 1 Imaging Sciences and Biomedical Engineering, King's College London, London, UK; 2 PhySense, Universitat Pompeu Fabra, Barcelona, Spain; 3 Centre for Medical Image Computing (CMIC), University College London, London, UK

## Background

Atrial fibrillation (AF) can be treated by catheter ablation to electrically isolate the pulmonary veins (PVs) from the left atrial (LA) body. To guide the procedure, an anatomical representation of the LA is usually visualised by an electroanatomical mapping system. As a follow-up, late gadolinium enhancement MR sequences can be obtained to assess the extent of ablation delivery. However, visual analysis and quantification is time-consuming with poor reproducibility. Unfold maps offer advantages over 3D surfaces for visualisation of imaging information while providing a common reference space for inter-subject comparison. We have developed a method to automatically map multimodal information onto a standardised unfold map (SUM) of the LA. We use the SUMs to compare force and scar information from the same patient.

## Methods

Ten patients underwent ablation procedure for AF. The intra-procedure anatomical surfaces containing force measurements from the CARTO system were exported. Six months after the procedure, the patients underwent a cardiac MRI scan in order to quantify the extent of post-ablation injury. The scan included 3D whole heart (3DWH) and 3D late enhancement (3DLE) sequences.

The LA was segmented from the 3DWH datasets using a multi-atlas fusion approach. The segmentations were manually corrected when necessary to generate a high fidelity result. The LA segmentation initialised a 3DLE scar extraction algorithm based on maximum intensity projection. We extracted a surface mesh from the LA segmentation and mapped the 3DLE intensity values to corresponding surface mesh locations. To standardise the resulting surfaces, we automatically clipped the mitral plane and the PVs 10mm distal to the ostia. To bring each mesh to the average mesh space, we computed a landmark based affine transformation using the PV end points and the mitral centroid. We applied this transformation to each patient's mesh and performed surface matching via currents. After surface registration, we projected the scar and force values of each patient onto the average mesh. The average mesh was unfolded using a fast surface parameterisation technique developed for texture mapping. The mapping method enables the constraint of the parameterisation to fit a predefined template.

## Results

**Figure 1:**
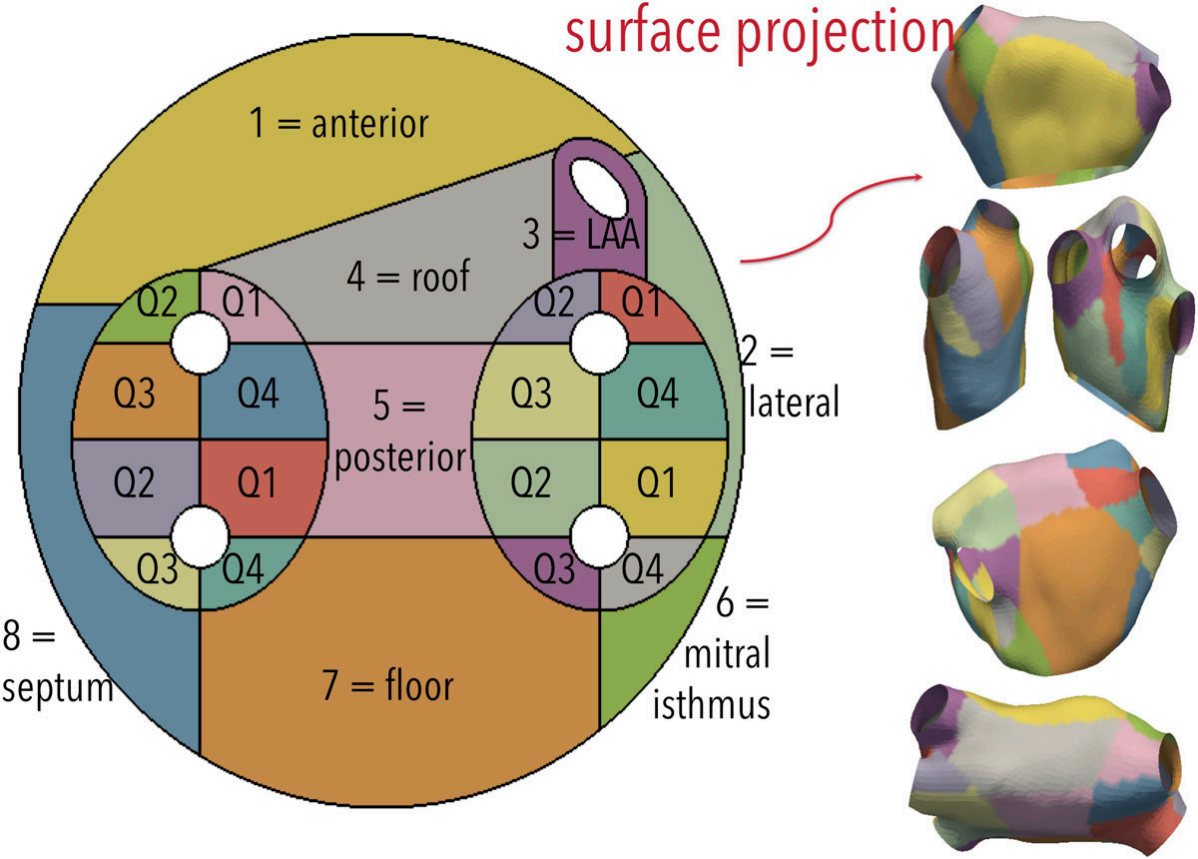
Proposed 24 region standard unfold map (left) and its corresponding visualisation in 3D (right)

**Figure 2:**
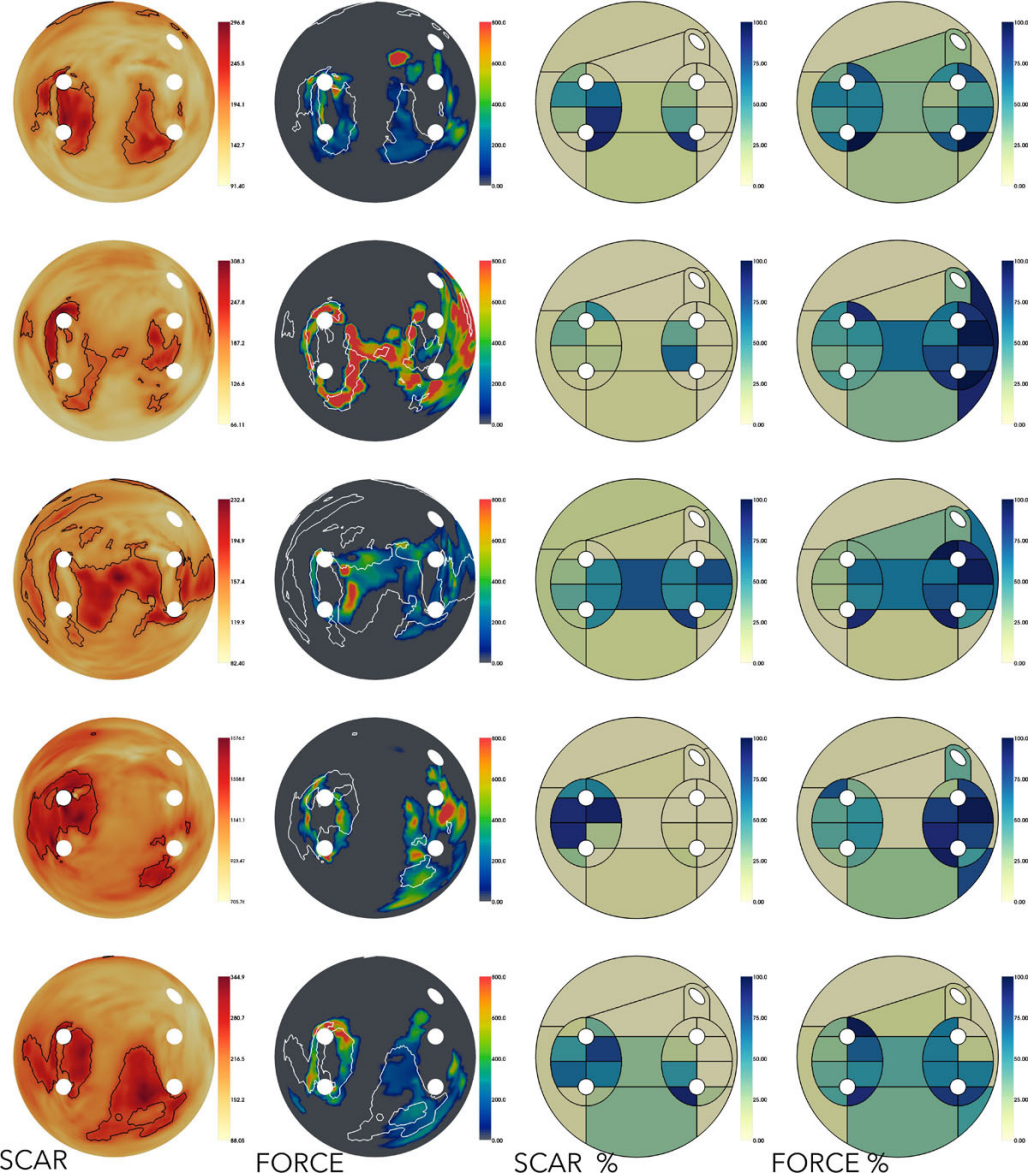
Each row shows the generated SUMs corresponding to one patient. COLUMN 1= 3DLE intensity values are shown in a yellow-red colormap. Black edges represent scar locations segmented with an expectation maximisation technique. COLUMN 2= force measurements are shown in a rainbow colormap. Gray means zero force. White edges represent scar locations obtained from the SCAR map (left). COLUMN 3= percentage of area covered by scar for each of the 24 regions. COLUMN 4= percentage of area covered by force (above zero) for each of the 24 regions.

The template SUM was split into 24 regions in accordance with published literature and the consensus of two experienced electrophysiologists (Fig. 1). We computed SUMs both from 3DWH-derived meshes and CARTO meshes. Using the regional division we automatically computed the percentage of post-ablation lesions around each PV (Fig. 2).

## Conclusions

This approach is robust to multiple types of input data and displays a unified holistic unfold from multimodal information. It also allows computing metrics per region in an automatic manner. Examples of metrics include: average force value, lesion area coverage, lesion circumferential coverage. The proposed SUM for the LA is analogous to the bulls eye plot for the left ventricle.

## Funding

This research was supported by the National Institute for Health Research (NIHR) Biomedical Research Centre at Guy's and St Thomas' NHS Foundation Trust and King's College London. The views expressed are those of the author(s) and not neces- sarily those of the NHS, the NIHR or the Department of Health.

